# Placenta increta presenting with threatened miscarriage during the first trimester in rhesus-negative mother: a case report

**DOI:** 10.1186/s13256-021-03030-x

**Published:** 2021-09-08

**Authors:** Nik Lah Nik-Ahmad-Zuky, Azmel Seoparjoo, Engku Ismail Engku Husna

**Affiliations:** 1 Department of Obstetrics & Gynaecology, School of Medical Sciences, Universiti Sains Malaysia, 16150 Kubang Kerian, Kelantan, Malaysia; 2 Department of Pathology, School of Medical Sciences, Universiti Sains Malaysia, 16150 Kubang Kerian, Kelantan, Malaysia; 3 Hospital Universiti Sains Malaysia, 16150 Kubang Kerian, Kelantan, Malaysia

**Keywords:** Placenta accreta, First trimester, Rh-negative, Hysterectomy

## Abstract

**Background:**

Placenta accreta is known to be associated with significant maternal morbidity and mortality—primarily due to intractable bleeding during abortion or delivery at any level of gestation. The complications could be reduced if placenta accreta is suspected in a patient with a history of previous cesarean delivery and the gestational sac/placenta is located at the lower part of the uterus. Then, a proper management plan can be instituted, and complications can be reduced. The diagnosis of placenta accreta in the first trimester of pregnancy is considered uncommon.

**Case presentation:**

A 34-year-old Malay, gravida 4, para 3, rhesus-negative woman was referred from a private hospital at 13 weeks owing to accreta suspicion for further management. She has a history of three previous lower-segment cesarean sections. She also had per vaginal bleeding in the early first trimester, which is considered to indicate threatened miscarriage. Transabdominal ultrasound revealed features consistent with placenta accreta spectrum. She was counseled for open laparotomy and hysterectomy because of potential major complication if she continued with the pregnancy. Histopathological examination revealed placenta increta.

**Conclusion:**

A high index of suspicion of placenta previa accreta must be in practice in a patient with a history of previous cesarean deliveries and low-lying placenta upon ultrasound examination during early gestation.

## Introduction

Placenta accreta refers to abnormal trophoblast invasion involving part or all of the placenta into the myometrium due to a defect in the deciduo–myometrial interface, leading to morbid adherence to the uterus and an inseparable placenta upon delivery [1]. The condition is associated with massive obstetric hemorrhage in both diagnosed and undiagnosed cases, but it is more catastrophic in the latter and carries significantly higher morbidity and mortality risks for the mother [2]. Placenta accreta spectrum (PAS) refers to the range of trophoblast and villous tissue invasions in the myometrium; placenta creta refers to the villi adhering to the myometrium; placenta increta refers to when the villi invade the myometrium; and placenta percreta refers to when the villi invade the full thickness of the myometrium [3]. PAS incidence is an increasing trend worldwide due to the increasing number of cesarean deliveries and concurrent placenta previa [4]. The incidence of placenta previa accreta was 4.1% in women with one previous cesarean delivery and 13.3% in women with more than two cesarean deliveries [5].

The overall prenatal ultrasound diagnosis of PAS in women with placenta previa and a history of cesarean delivery is 90.9%. Most diagnoses are made during the second and third trimesters of pregnancy [5]. A first-trimester diagnosis of PAS is considered rare and challenging [6, 7]. The classical signs or clinical diagnosis of placenta accreta such as demonstration of placental lacunae, loss of the clear zone, bladder wall interruption, and uterovesical hypervascularity can be identified via ultrasound starting at 11–14 weeks of pregnancy [8]. In patients with risk factors for placenta accreta who have a first-trimester ultrasound examination showing signs of PAS, it is important to thoroughly discuss the treatments options with the patients, with the aim of generating the fewest possible complications. Here, we describe a case of a patient 13 weeks pregnant who had three previous cesarean deliveries, was Rh-negative, and diagnosed with placenta increta.

## Case presentation

A 34-year-old Malay, gravida 4, para 3, Rh-negative woman was referred from a private hospital at 13 weeks owing to accreta suspicion for further management. She had a history of three previous lower segment cesarean sections, and all operations were uneventful. At 5 weeks of pregnancy, she presented with per vaginal bleeding and unresolved suprapubic pain at a private hospital. Her urine pregnancy test was positive, and ultrasound examination showed an empty uterus with evidence of intraperitoneal bleeding. A diagnosis of a ruptured ectopic pregnancy was made. She underwent emergency laparotomy, and hemoperitoneum with clots and fresh 500 ml of bleeding were found. This was due to bleeding from a ruptured vessel of an engorged and swollen left Fallopian tube. Left salpingectomy was performed. Postoperatively, her per vaginal bleeding had stopped, and, on day 3 postoperation, she was discharged from the ward. A week later, she had had obvious morning sickness symptoms; she then returned to her doctor and discovered she had an intrauterine pregnancy with a viable fetus of 7 weeks gestation.

**Fig 1:**
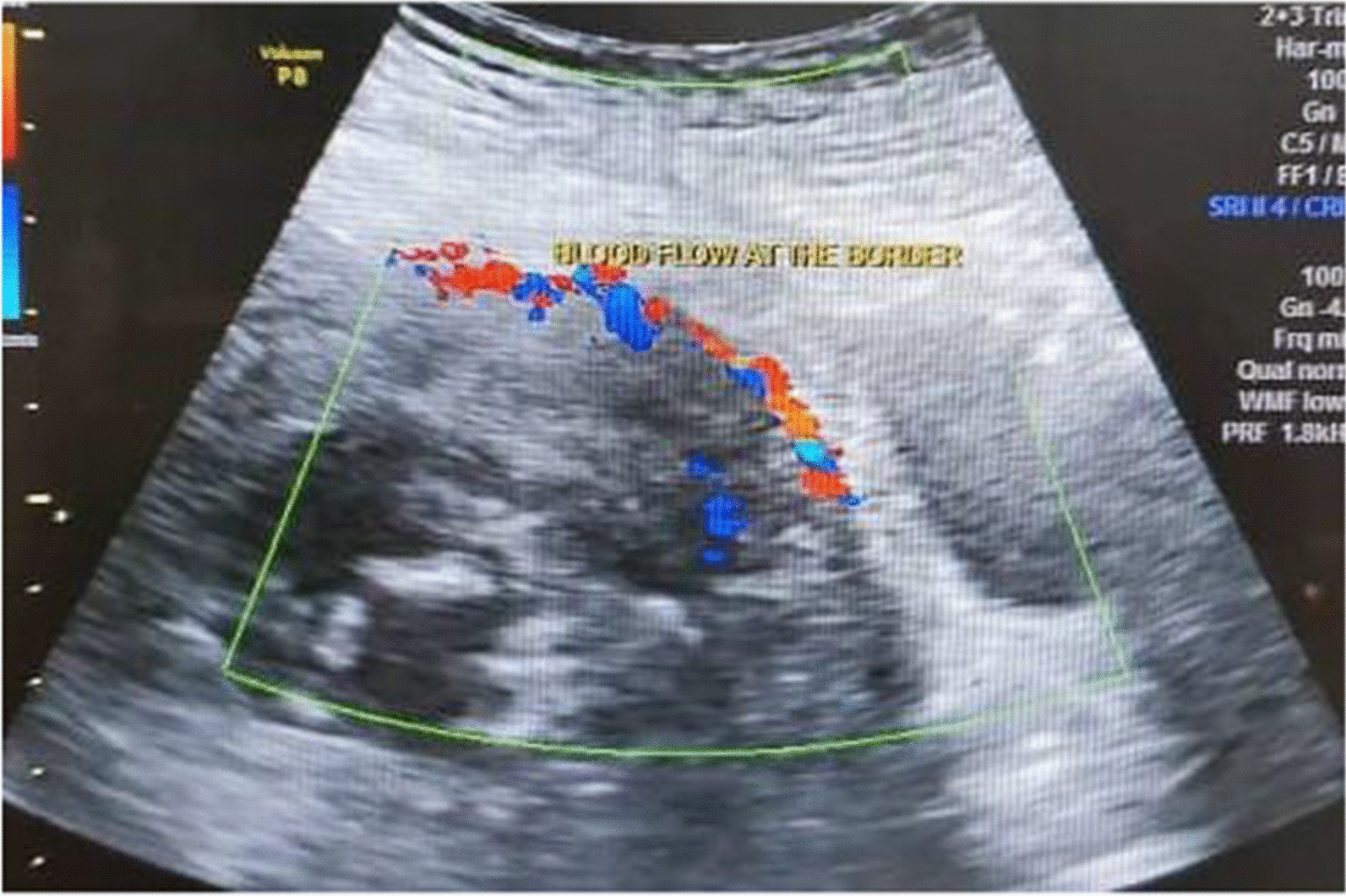
Transabdominal ultrasound showed an increase of blood flow between the uterus and urinary bladder border

The gestational sac was located at the lower part of the uterus; however, there was no suspicion of abnormal placentation at that time. The patient was given 4 weeks until her next appointment. She experienced intermittent minimal per vaginal bleeding associated with suprapubic discomfort during this period. At 12 weeks of gestation, a repeat ultrasound showed that a viable fetus was located at the lower part of the uterus, and the placenta was covering the internal os, which was accompanied by loss of the hypoechoic border between the placenta and uterus; thus, a diagnosis of placenta accreta was made. The patient sought a second opinion from another consultant. Magnetic resonance imaging (MRI) was performed, and the gestational sac was found to occupy the lower half of the uterine cavity. Moreover, superior to the gestational sac was a sizeable heterogeneous lesion, suggestive of a multi-age blood clot occupying the other half of the uterine cavity. The placenta was located at the lower part of the uterus covering the os. She was counseled for a hysterectomy and then was referred to our center. A repeat ultrasound examination revealed similar findings with increased subplacental vascularity at the uterine bladder interface (Fig.1). Per-abdominal examination revealed that the uterus was at 20 weeks gravid uterine size.

An elective hysterectomy was decided upon, and the procedure and possible complications were explained to the patient and partner. The patient was started with an intravenous antibiotic because of her prolonged per vaginal bleeding. The challenge in managing the case was in deciding the best approach to minimize the patient’s complications. A large amount of Rh-negative blood is not readily available in our blood bank. If an additional amount is required, a regular donor needs to be called, or Rh-negative blood is collected from another hospital blood bank. The surgery could only be performed after at least 6 pints of blood group O Rh-negative was obtained in preparation for any bleeding intraoperatively. The apprehension was more regarding the adhesion of the uterus to the anterior abdominal wall, the difficulty of separating the urinary bladder, the possible injury to the urinary bladder, and intraoperative bleeding. The transfusion department of our institution managed to gather eight units of a packed cell of Rh-negative blood group O on the operation day. The urology team was on standby during the operation. A midline subumbilical vertical incision was made. There were adhesions between the right anterolateral peritoneal wall with the omentum, the anterior surface of the uterus, and the bowels. Adhesiolysis was carried out slowly.

**Fig 2:**
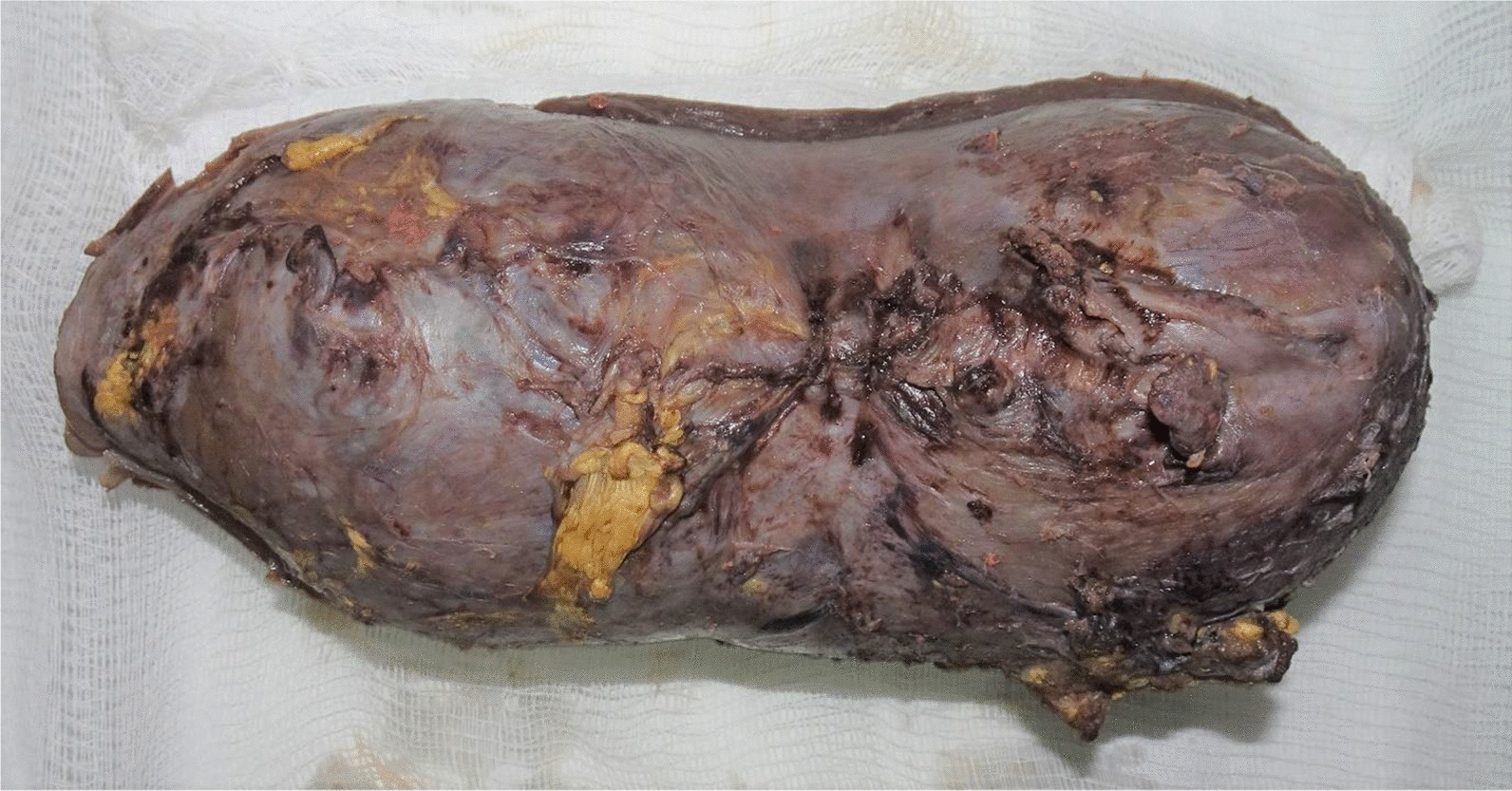
Gross specimen of the uterus with evidence of adhesion over the anterior uterine serosal surface

**Fig 3:**
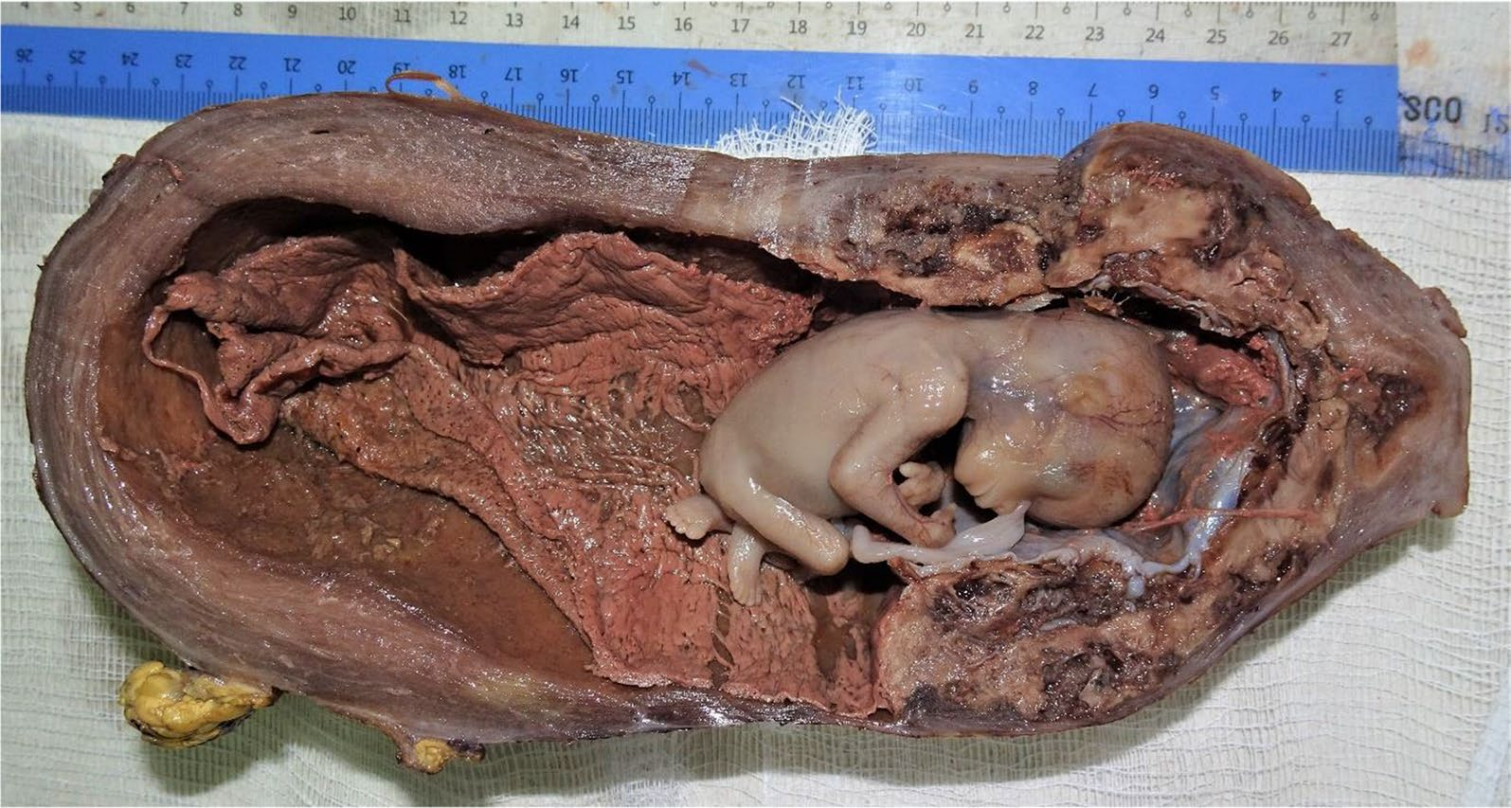
Gross specimen with well-formed fetus with attached umbilical cord. The placenta invades the myometrium but does not breach the serosa lining. The upper part of uterus was filled up by blood clots

**Fig 4:**
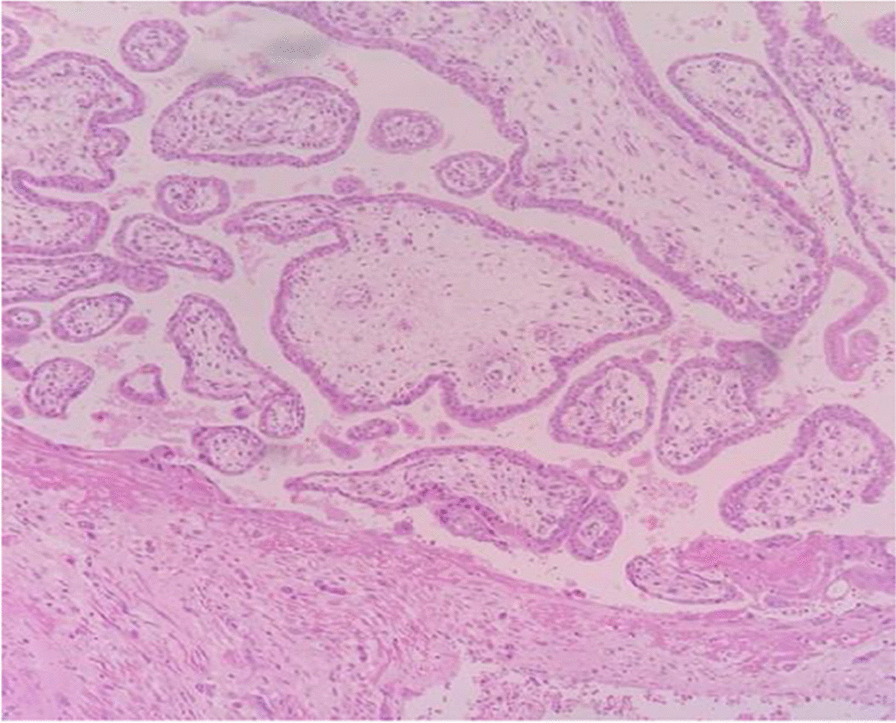
Chorionic villi seen invading into the myometrium with absence of decidual tissue

An enlarged uterus was visible, with tortuous vessels on the serosal surface of the lower part. The total hysterectomy was performed successfully. The estimated blood loss was 2 L, with bleeding mainly from the raw areas at the vesicouterine fold. Two pints of the packed cell were transfused intraoperatively. A gross histopathological examination showed that the placenta appeared to extend up to the serosa (Figs. 2, 3). It was microscopically confirmed that the chorionic villi invaded the myometrium with an absence of decidual tissue, while no invasion toward or penetration of the serosal layer was found (Fig. 4). Our patient recovered uneventfully. She was discharged on the fifth postoperative day in good condition, and she was in excellent health during a follow-up visit 2 weeks later. She was seen again after 1 month: she had no complaints, the wound was healed, and she was discharged from the gynecological clinic.

## Discussion

A high index of suspicion of placenta previa accreta during early gestation must be in practice in patients who have a history of previous cesarean deliveries and the gestational sac/placenta located at the lower part of the uterus upon ultrasound examination [6]. Ultrasound examination should be repeated at 2-week intervals, preferably with a transvaginal approach if, upon initial examination, an accreta has not been excluded. Ultrasound findings suggestive of accreta in the first trimester are similar to those of the second or third trimester [8]. Our case showed a loss of the hypoechoic border between the placenta and uterus and increased subplacental vascularity at the uterine bladder interface. MRI examination of the uterus and placenta is reserved for those with inconclusive findings upon ultrasound examination [9].

A prolonged threatened miscarriage in the first trimester in patients in the high-risk group should also be carefully regularly evaluated. The early diagnosis of accreta is crucial for a patient to be given the option to continue with their pregnancy, but, typically, patients choose hysterectomy or to terminate the pregnancy with a high chance of uterine preservation. Most of the literature describes a clinical diagnosis of placenta accreta during or after suction dilatation and curettage due to first-trimester miscarriage [7]. In most cases, the procedure was complicated with profuse bleeding, and most end up with emergency hysterectomy [7].

A planned transabdominal hysterectomy is the definitive treatment for the patient. It was considered to be the best option, as uterine preservation was not an issue. Uterine preservation might have been considered if the diagnosis had been made much earlier. Then, a surgical termination with ultrasound-guided suction and curettage could have been carried out with minimal risk of maternal mortality [10].

In a prospective study of 22,604 women who underwent an ultrasound scan at 11–13 weeks, 1256 of them were considered as high risk due to their having both previous uterine surgery and a low-lying placenta, while only 14 of them were diagnosed with accreta. Among the high-risk group, the incidence of accreta increased with advancing gestational age owing to decreasing incidence of a low-lying placenta, from 1% at 14 weeks to 6% at 22 weeks and 27% at 30 weeks. All 14 women with accreta chose to continue with their pregnancies to delivery after extensive counseling concerning the potential complications during both pregnancy and delivery [10]. As shown in our case, Rh-negative blood is not readily available in large amounts at our institution. Individuals with Rh-negative blood account for less than 0.2% among the Malaysia population. Based on reported data, the mean blood loss during surgery was 4.0 (range 1.5–22.0) L [10]. The patients should not be exposed to uterine rupture during the second trimester before fetal viability, thus avoiding maternal mortality or severe morbidity.

Common iliac artery balloon occlusion (CIABO) as an adjunct to the surgery might be thought by some to reduce intraoperative bleeding, as it appeared to reduce intraoperative blood loss during hysterectomy significantly [11]. It was less favorable in her case because of her relatively small uterine size and the incision of the uterus not being required. Besides, complications related to CIABO procedure such as arterial thrombosis could be avoided.

## Conclusion

A high index of suspicion of placenta previa accreta during early gestation must be in practice in patients with a history of previous cesarean deliveries and a low-lying placenta upon ultrasound examination. The management must be appropriately planned to reduce complications as much as possible. The patient and their partner must also be counseled in detail since the outcome is not always as expected.

## Data Availability

The patient’s information and medical records used for the case report are available from the corresponding author upon request.

## Abbreviations

CIABO: Common iliac artery balloon occlusion
Rh: Rhesus
